# The Isolation of Specialty Compounds from *Amphidinium carterae* Biomass by Two-Step Solid-Phase and Liquid-Liquid Extraction

**DOI:** 10.3390/toxins14090593

**Published:** 2022-08-28

**Authors:** Mercedes López-Rodríguez, Lorenzo López-Rosales, Giullia Diletta, María del Carmen Cerón-García, Elvira Navarro-López, Juan José Gallardo-Rodríguez, Ana Isabel Tristán, Ana Cristina Abreu, Francisco García-Camacho

**Affiliations:** 1 Department of Chemical Engineering, University of Almeria, 04120 Almeria, Spain; 2 Research Centre CIAIMBITAL, University of Almeria, 04120 Almeria, Spain; 3 Department of Chemistry and Physics, University of Almeria, 04120 Almeria, Spain

**Keywords:** dinoflagellate, solid-phase extraction, liquid-liquid extraction, amphidinol, fatty acids, carotenoids

## Abstract

The two main methods for partitioning crude methanolic extract from *Amphidinium carterae* biomass were compared. The objective was to obtain three enriched fractions containing amphidinols (APDs), carotenoids, and fatty acids. Since the most valuable bioproducts are APDs, their recovery was the principal goal. The first method consisted of a solid-phase extraction (SPE) in reverse phase that, for the first time, was optimized to fractionate organic methanolic extracts from *Amphidinium carterae* biomass using reverse-phase C18 as the adsorbent. The second method consisted of a two-step liquid-liquid extraction coupled with SPE and, alternatively, with solvent partitioning. The SPE method allowed the recovery of the biologically-active fraction (containing the APDs) by eluting with methanol (MeOH): water (H_2_O) (80:20 *v*/*v*). Alternatively, an APD purification strategy using solvent partitioning proved to be a better approach for providing APDs in a clear-cut way. When using n-butanol, APDs were obtained at a 70% concentration (*w*/*w*), whereas for the SPE method, the most concentrated fraction was only 18% (*w*/*w*). For the other fractions (carotenoids and fatty acids), a two-step liquid-liquid extraction (LLE) method coupled with the solvent partitioning method presented the best results.

## 1. Introduction

Marine microalgae are a promising feedstock replete with fascinating bioactives. However, industrial-scale production of high-value bulk commodities is still a long way off, unless both bulk and specialty co-products can be obtained from the cultivated microalgae; only in this way can the production of bulk commodities become economically viable [1]. Species from classes, dinoflagellate and raphidophyceae, are known to produce metabolites with interesting bioactivities. For example, *Amphidinium carterae* is a dinoflagellate microalgae producer of amphidinols (APDs). In the context of biorefining dinoflagellate microalgae, the APD targeted approach, based on recovering the bioactive compounds from the *Karlodinium* cultures described [2], was used to extract APDs and assess the recovery of the three families of compounds from *A. carterae*, namely polyunsaturated fatty acids (PUFAs), carotenoids, and APDs [3]. The overall APD recovery obtained in that study was almost 70%, the main enriched fractions being MeOH-H_2_O (80:20 *v*/*v* and 60:20 *v*/*v*) with 33% and 13% of the APDs present in the crude extract, respectively. Although the results of that study were initially successful, around 11% of the APDs were not adsorbed on the column due to the quantity of biomass being too high for the sorption capacity of the cartridges used. In contrast, an integrated bioactive compound-targeted approach has proven suitable for readily separating APDs, carotenoids, and fatty acids contained in *A. carterae* biomass [4]. In that study, an APD–carotenoid-targeted process involving a fractionation step using solid-phase extraction (SPE) provided two distinct fractions that were quite APD-enriched, with no metabolites detected after passing the extract through the reverse-phase C18 solid phase. Although high APD recoveries were obtained, the process did not achieve clear-cut APD purification in which the fractions had no interferences from other metabolites. Indeed, the nuclear magnetic resonance (NMR) analysis showed tiny amounts of compounds, namely organic acids, which were eluted in these fractions. In addition, a small percentage (almost 8%) of the APDs was detected in the 100% water elution fraction.

SPE is a general technique for separating, concentrating, and purifying crude extracts from complex matrices. It is widely applied as a fractionation step for multiple purposes, including purification, trace enrichment, and class fractionation, among others [5,6]. This technique involves three main steps: sample preparation, column equilibration and retention, and the elution gradient. The sample volume is one of the primary factors that determines the retention capacity of the analyte sorbent (milligrams of analyte per gram of sorbent) [6,7]. A useful parameter for characterizing an SPE device is the breakthrough volume (V_B_), which is established from the breakthrough curve, i.e., the point on the curve where some arbitrary amount of sample is detected at the outlet of the sampling device during the retention step [8]. In brief, the concentration of the solute in the effluent begins to increase at a starting point that is denoted as the breakthrough volume (V_B_). The corresponding concentration for measuring V_B_ is usually taken as the minimal percentage of the initial analyte concentration (*C*_0_). As the solution continues to pass through the sorbent, the value of the effluent concentration of the analyte (*C*) tends to its maximum, reaching the point of inflection that is denoted as the retention volume (V_R_). Therefore, V_R_ is defined as the maximum loading capacity of the column [6,8]. The extracts from *A. carterae* biomass comprise a complex matrix containing a large amount of potentially interfering compounds that are generally present at higher concentrations than other relatively minor compounds, such as APDs.

Liquid-liquid extraction (LLE) is widely used to separate target compounds; it is based on the relative solubilities of these compounds in two different immiscible liquids, typically water and organic solvents [9]. As a prior isolation step, the use of a suitable organic solvent (determined by the analyte’s partition coefficient) can be an effective tool for subsequent SPE purification procedures [10,11]. In addition, LLE has been used as a method for sample pre-concentration and purification to detect marine toxins, given their expected concentration and the complexity of the samples [9,12].

The present work focuses on optimizing an SPE-based method using C18 as the reverse-phase adsorbent to select and effectively fractionate extracts from *A. carterae;* this is done to obtain a rough separation of the three families of bioactive compounds. Since APDs are the main target, the process uses methanol–water mixtures as the eluent in an increasing order of polarity, as well as combining solid-liquid extraction as a pre-treatment followed by solid-phase extraction. The first step aims to optimize the biomass cell disruption and then to optimize the biomass-to-extractant ratio to obtain the maximum amount of APDs. The following determination of the breakthrough volume provides the adsorbent-to-biomass extract ratio, which ensures the adsorbent’s maximum adsorption capacity of the organic metabolites. The process was initially scaled to a 10-g C18 cartridge and then scaled up to an 80-g C18 column using two approaches: (i) direct fractionation of the crude extract and (ii) LLE based on organic solvent extraction and subsequent two-way purification—by SPE and by solvent partitioning with n-butanol (BuOH)—as an APD-prioritized fractionation strategy to develop a biorefinery procedure for the integral valorization of this dinoflagellate. The flowsheet of the processes undertaken is shown in the graphical abstract.

## 2. Results and Discussion

### 2.1. Evaluation of Biomass Pre-Treatments and Extraction Optimization

**Figure 1:**
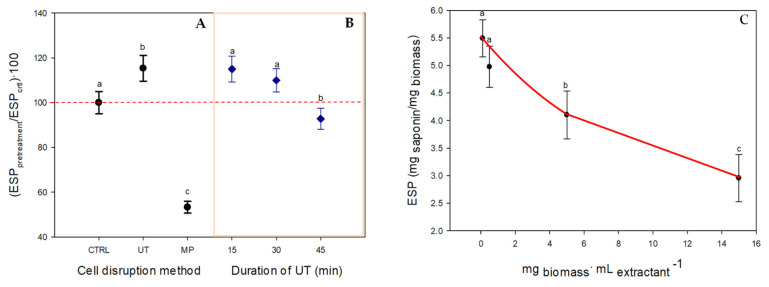
(**A**) Effect of different cell disruption methods on the extraction of APDs from *A. carterae* biomass, expressed in terms of hemolytic activity relative to the control. CTRL: control; UT: ultrasound; MP: mortar and pestle without alumina. (**B**) Effect of the ultrasound time (min) on the extraction of APDs. (**C**) Influence of the biomass-to-extractant ratio on hemolytic activity (ESP: equivalent saponin potency) of methanolic extracts produced from *A. carterae* biomass and treated with UT for 15 min. Data points are averages and vertical bars are the standard deviations for triplicate samples. The lowercase letters represent significant differences, with a *p*-value < 0.05.

The optimization of the extraction procedure to determine the APDs was carried out by considering the cell disruption method, the pre-treatment duration, and the crude extract concentration (see Graphical Abstract and Section 4.2.2 and Section 4.2.3 for more details). Figure 1A compares the effectiveness of the two cell breakage methods tested in terms of the hemolytic activity related to the content of APDs [13]. The relative hemolytic activity, expressed as the equivalent saponin potency of the extract obtained after the pre-treatment (ESP_pretreatment_) relative to control ESP_crtl_, was observed with all three cell disruption methods: ultrasound (UT), the absence of a pre-treatment (CTRL), and milling (MP) (Figure 1A). UT was the most efficient disruption method, followed by the control (CRTL) and MP (in decreasing order). UT has been widely used for rupturing microalgae cells, particularly to improve the extraction of these types of secondary metabolites from dinoflagellates, the structures of which seem to play a role in binding to the lipid bilayer membrane [14]. Combining UT with methanol as the solvent has also proven to be a very efficient extraction method in various biological systems [5,15]. An earlier study using *A. carterae* biomass compared the effectiveness of different cell breakage methods in terms of their carotenoid and fatty acid recovery [16]. In that study, although UT was the second most suitable method for recovering carotenoids, it recovered relatively low amounts of fatty acids. In our study, UT significantly increased the hemolytic activity compared to the CTRL, indicating that cell disruption methods are necessary for APD recovery. The ESP value remained constant for sonication times from 15 to 30 min (*p*-value < 0.05) (Figure 1B), indicating that maximum APD extraction was achieved after 15 min of treatment. Conversely, the ESP value decreased for sonication times under 45 min. Therefore, 15 min was used for the rest of the study.

The extraction of microalgal bioproducts was chiefly conducted using dried biomass treated with organic or aqueous solvents, depending on the polarity of the target compound [17]. With regard to the APDs, their maximum extraction takes place at polarity indexes and solubility parameter values that are close to methanol (above 6 and 20 MPa^1/2^, respectively) [16]. In addition, sample preparation requires the optimal quantity of biomass to be established in order to achieve a preliminary characterization of these minority metabolites compared to the carotenoids and fatty acids. As can be seen in Figure 1C, a greater hemolytic power (ESP = 5.49 mg _saponin_·mg _biomass_^−1^) was reached at the lowest biomass-to-extractant ratios of 0.1 and 0.5 mg·mL^−1^ (equivalent to 0.06 mg _extract_·mL^−1^ and 0.3 mg _extract_·mL^−1^ in the hemolytic activity assay, respectively). Above 0.5 mg·mL^−1^, the ESP decreased as the biomass-to-extractant ratio increased, with the lowest ESP value (2.96 mg _saponin_·mg _biomass_^−1^) being reached at a ratio of 15 mg·mL^−1^. In this respect, as explained elsewhere [18], high biomass-to-extractant ratios imply high analyte concentrations in the extract, which might compromise the selectivity and cause a drop in sensitivity for the analyte of interest in the sample matrix [18].

### 2.2. Evaluation of Breakthrough Curves

The maximum amount of material that can be retained in an SPE device must be optimized to ensure a theoretical retention percentage of 95–99% [19]. This is known as the breakthrough volume, which depends on the concentration of analytes in the solution loaded onto the sorbent. For this, the sample concentration that can be loaded onto the sorbent bed must be optimized. One approach to determine the retention capacity is the equilibrium method, in which a given volume of sample solution with a known concentration of analyte is circulated through the SPE device until a steady state is reached [20]. A lack of retention on an SPE device can be caused by the addition of too much mass in the load. In this scenario, analytes are not quantitatively retained by the sorbent and are thus detected in the effluent (the unretained volume sample) [6]. With the goal of minimizing the loss of APDs in the effluent fraction (a maximum of 5% relative to the loaded crude extract amount), the retention capacity of the 1-g reverse-phase C18 cartridge was evaluated, as detailed in Section 4.2.4 below.

**Figure 2:**
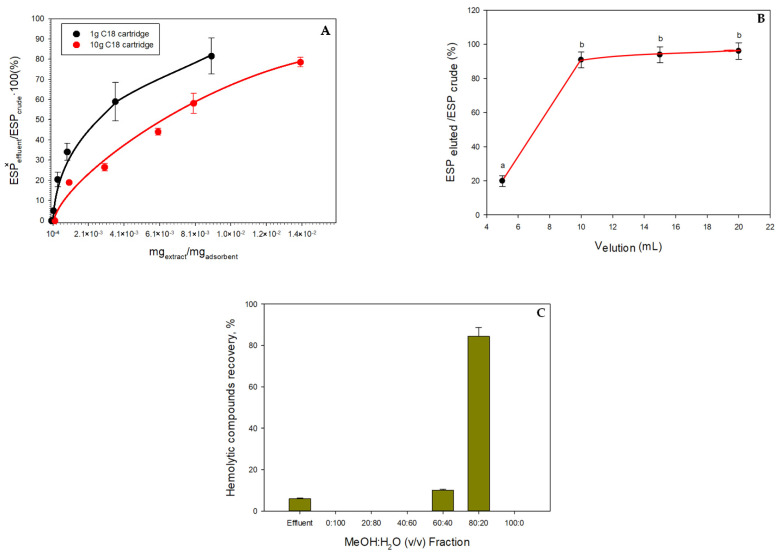
(**A**) Determination of the breakthrough curves in terms of the ESP_effluent_-to-ESP_crude_ ratio versus the extract-to-adsorbent ratios for 1-g reverse-phase C18 cartridges (black points) using crude MeOH extracts, and for 10-g reverse-phase C18 cartridges (red points) using an extract from the clear phase obtained in Figure 4, Step 2. (**B**) Optimization of the elution volume with 100% MeOH to completely elute all the compounds adsorbed. (**C**) Distribution of the hemolytic compounds recovered in the fractionation process using a 1-g reverse-phase C18 cartridge. The recovery of hemolytic compounds was calculated from the ESP_eluted_-to-ESP_crude_ measurements. Data points and bars are averages and vertical bars are standard deviations for triplicate samples. The lowercase letters in Figure 2B represent significant differences, with a *p*-value < 0.05.

Figure 2A displays the breakthrough curves obtained, represented as the ESP_effluent-_to-ESP_crude_ ratio—defined in Section 4.2.4—against the amount of crude MeOH extract and clear phase that were loaded in the 1-g and 10-g C18 cartridges (expressed in terms of the crude extract-to-adsorbent and clear phase extract-to-adsorbent ratios), respectively. One can observe that APD losses in the effluent (i.e., the ESP_effluent_-to-ESP_crude_ ratio) above 5% appeared at crude extract-to-adsorbent and clear phase extract-to-adsorbent ratios above 1.20 × 10^−4^ and 2 × 10^−4^ (mg _extract_·mg _adsorbent_^−1^) for the 1-g and 10-g C18 cartridges, respectively (Figure 2A). APD losses in effluents were reported in a previous study [3]; the authors concluded that they might be due to the quantity of biomass used being too high. In our findings, only about 5% of losses (in terms of ESP) were detected when a crude extract-to-adsorbent ratio of 1.20 × 10^−4^ was used, whereas about 80% were detected at the highest ratios (Figure 2A). Typical chemically-bonded sorbent has a capacity of about 1–10% of their weight [6]. In some cases, the sorbent bed fails to retain the target compound during the charge step or even during washing. Complex matrixes where other majority metabolites also bind to sorbent can cause this. Concretely, *A. carterae* biomass crude extracts contain a large number of compounds that are generally present at higher concentrations than APDs [3]. In this respect, to retain the largest amount of the target metabolites, a crude extract-to-adsorbent ratio of 1.20 × 10^−4^ was selected.

### 2.3. Optimization of the Elution Volume

With the goal of desorbing the entire mass of the metabolites (particularly the APDs) retained on the C18 bed, 1-g C18 cartridges were eluted with different volumes of MeOH (100%). The eluted solutions were collected, and their hemolytic activity was measured (see Section 4.2.4). Figure 2B shows the results obtained, indicating that a 10-mL volume of MeOH was sufficient to desorb more than 90% of the target compounds. There were no statistically significant differences between using 10, 15 or 20 mL of MeOH (*p*-value < 0.05). Therefore, 10 mL was chosen as the elution volume.

### 2.4. Evaluation of SPE-Based Fractionation

**Figure 3:**
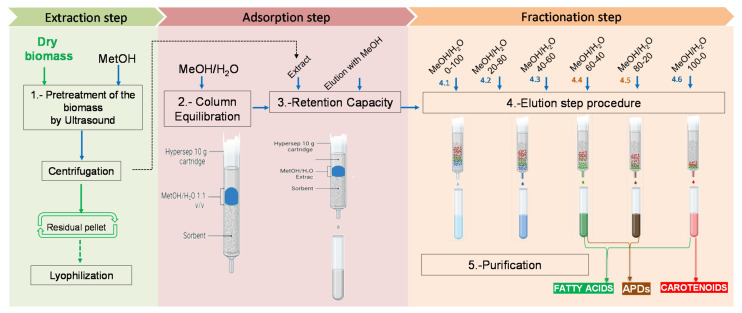
The direct fractionation by SPE approach using 1–10-g reverse-phase C18 cartridges.

Based on the previous results, a crude MeOH extract volume corresponding to the crude extract-to-adsorbent ratio of 1.20 × 10^−4^ (*w*/*w*) was loaded into a 1-g C18 cartridge (see the adsorption step in Figure 3) and subjected to a six-step elution protocol, as described in Section 4.2.5 (see the fractionation step in Figure 3). The results are presented in Figure 2C. Hemolytic activity was only observed in the fractions obtained by eluting with MeOH:H_2_O 80:20 (*v*/*v*) and MeOH:H_2_O 60:40 (*v*/*v*). The ESP value of the MeOH:H_2_O 80:20 (*v*/*v*) fraction was 7 ± 0.35 mg _saponin_·mg _extract_^−1^, which is equivalent to 84.35 ± 4.21% of the APDs in the crude extract (Figure 2C)—whereas the MeOH:H_2_O 60:40 (*v*/*v*) fraction contained 10 ± 0.5% of APDs (equivalent to 0.83 ± 0.04 mg _saponin_·mg _extract_^−1^). A similar distribution pattern was observed in previous studies [3,4], but with an APD recovery percentage of around 70% [3]—a value lower than that achieved in this study (94.35 ± 4.71%). Furthermore, the APD losses were only detected in the effluent, accounting for around 5% of the amount of APDs contained in the crude MeOH extract (see Figure 2C)—in contrast to the 11% reported elsewhere [3]. A few significant differences between our work and the previous studies mentioned above [3,4] seem to be the reason for this discrepancy: the extract carrying the APDs consisted of a hydroethanolic phase that might have contained traces of lipids, amino acids, sugars, or other compounds, and the biomass-to-adsorbent ratio was not optimized accurately [3,4]. However, an NMR analysis showed that the fraction with the APDs contained tiny amounts of amino acids (AA), organic acids (OA), polyhydric alcohols (PA), and sugar (SA). Indeed, the APDs were eluted between MeOH fractions that ranged from 60% to 100% MeOH, with no appreciable concentration pattern.

### 2.5. Scale-Up of the SPE to Obtain APDs, Carotenoids and Fatty Acids

**Figure 4:**
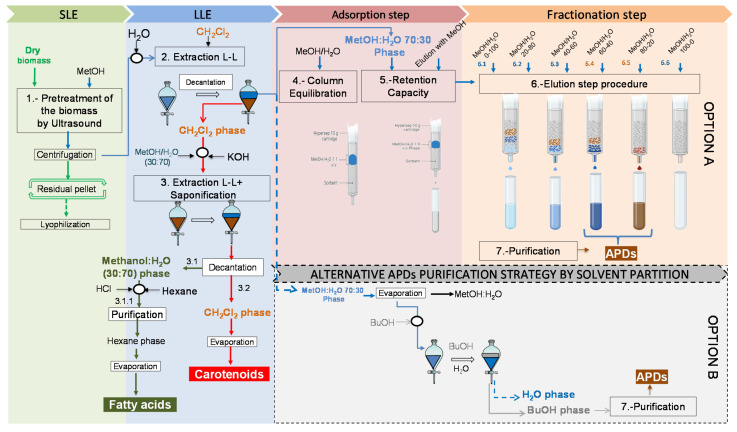
Flowsheet of the proposed process based on liquid-liquid extraction coupled with the SPE approach (Option A) using a reverse-phase 10-g C18 cartridge and 80-g C18 column, and an alternative APD purification strategy by solvent partitioning (Option B).

The results above were used to scale up (10-fold and 80-fold) the amounts of extracted biomass and C18 adsorbent. As explained in Section 4.3 of Materials and Methods, two approaches were evaluated for minimizing the target compound losses in the different fractions and for improving the APD purification, providing a clear-cut fraction with no interferences from other metabolites. These approaches were: (i) direct fractionation by SPE (see Figure 3) and (ii) liquid-liquid extraction (LLE) coupled with SPE, including an alternative purification step involving solvent-partitioning (see Figure 4).

#### 2.5.1. Evaluation of the Direct Fractionation by SPE Approach

Section 4.3.1 describes this approach and Figure 3 shows a scheme for the process. A 12-mL volume of crude MeOH extract (0.3 mg·mL^−1^) from 0.5 mg_biomass_·mL^−1^ extracted with methanol was mixed with 108 mL of H_2_O to obtain a 10% MeOH concentration. The final hydromethanolic extract was fractionated following the previous optimizations. This volume of crude MeOH extract was intentionally selected based on the data from the rupture curves displayed in Figure 2A. This volume involved APD losses of around 20% relative to the crude MeOH extract. The reason for the increase in APD losses in the effluent from 5% (as explained in Section 4.2.4) to 20% relates to the detection limit of the NMR analysis for the different polar metabolites that are potentially present in the collected fractions. To characterize the crude MeOH extract, a 41-mL sample was analyzed using the NMR-based metabolomics approach (see Section 4.5) to determine the APD content as well as other polar metabolite classes (i.e., amino acids, AA; organic acids, OA; sugars, SA; quaternary ammonium compounds, QAC; polyhydric alcohols, PA; and nitrogenous bases, NB).

**Figure 5:**
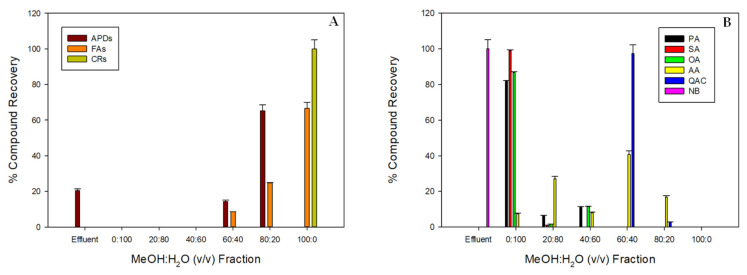
(**A**) Percentage distribution of the three metabolite groups and (**B**) the predominant groups of polar metabolome components in the fractionation step (direct fractionation by SPE) outlined in Figure 4, using a 10-g C18 cartridge. APDs: amphidinols; FAs: Fatty acids; CRs: Carotenoids; AA: amino acids; OA: organic acids; SA: sugars; QAC: quaternary ammonium compounds; PA: polyhydric alcohols; NB: nitrogenous bases. Percentages are relative to the content in the initial crude methanolic extracts.

The results of this approach are shown in Figure 5. The percentage distribution of the three main metabolite groups (APDs, carotenoids, and fatty acids) represented in Figure 5A demonstrates that APDs were separated in the MeOH–H_2_O (80:20 *v*/*v*) and MeOH–H_2_O (60:20 *v*/*v*) fractions with recovery yields of 65.25 ± 3.26% and 14.33 ± 0.72%, respectively. As expected, the APD losses in the effluent accounted for 20.42 ± 1.21%. The distribution of the remaining polar metabolites analyzed by NMR can be observed in Figure 5B. The OA, PA, and SA groups were swept along on the most polar fraction. NB was completely recovered in the effluent. The QAC group was concentrated in the 60:40 fraction. The AA group was detected in all the fractions, except for the 100:0 fraction with no clear distribution pattern. AA were found in a significant proportion in the most enriched APD stream (MeOH-H_2_O 80:20 *v*/*v*), along with a tiny percentage of QAC. All the carotenoids (100%) were found in the 100% MeOH fraction. The fatty acids were concentrated in the fractions from 60:40 to 100:0 (Figure 5A). This pattern was also observed by other authors [5], where it was explained that polar lipids, including glycolipids, were eluted in the same fraction as the APDs. This may be caused by the interactions between lipids and APDs mentioned above [14], which might enhance the APD desorption in the 60:40 fraction. These results indicate that some clean-up steps coupled with the solid-phase step are necessary to improve the procedure, as has been suggested [21,22,23].

#### 2.5.2. Evaluation of the Liquid-Liquid Extraction Coupled with SPE Approach

**Figure 6:**
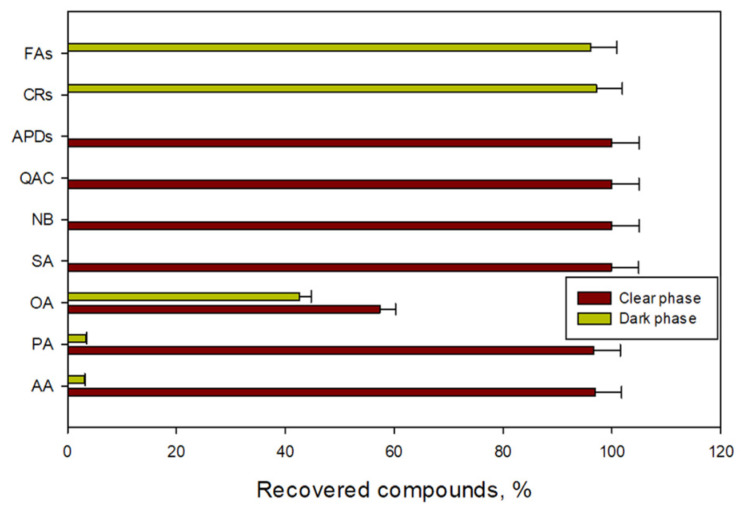
Distribution of the main metabolite groups throughout the two phases formed (the clear phase; 70:30 *v*/*v* MeOH: H_2_O, and the dark phase; CH_2_Cl_2_) following the liquid-liquid extraction of the crude methanolic extract outlined in Figure 4, Step 2. APDs: amphidinols; FAs: fatty acids; CRs: carotenoids; AA: amino acids; OA: organic acids; SA: sugars; QAC: quaternary ammonium compounds; PA: polyhydric alcohols; NB: nitrogenous bases. Data bars are averages and vertical bars are standard deviations for duplicates samples.

Section 4.3.2 explains the methodology that was followed to carry out this approach, and the scheme of the process is illustrated in Figure 4. The LLE step was applied to the crude MeOH extract. After the MeOH extract was extracted by LLE and separated by decantation (Figure 4, Step 2) and two phases were formed (the LLE step in Figure 4): the clear phase (i.e., the 70:30 *v*/*v* MeOH: H_2_O phase) and the dark phase (i.e., the CH_2_Cl_2_ phase). Figure 6 displays the distribution patterns of the recovery percentages of the three main compound families (APDs, fatty acids, and carotenoids) for both phases as well as the other polar metabolites. As expected, the carotenoids and fatty acids were swept along with the dark phase (δ_T_ 20 MPa^1/2^) (Figure 6) while the APDs were found in the clear phase (δ_T_ 34.83 MPa^1/2^), as well as the AA, PA, SA, NB, and QAC at percentages close to 100%. The OA distribution pattern was divided between the two phases. Based on this data, an enriched-APD fraction was obtained, making the subsequent fractionation step feasible.

For the APD determination, a 120-mL sample from the clear phase (MeOH:H_2_O 70:30 *v*/*v*), corresponding to 0.1 mg _clear phase extract_·mL^−1^, was dried and analyzed using an NMR-based metabolomics approach for the same purpose as that explained in Section 2.5.1. The breakthrough volume was determined by loading different volumes of clear phase, which corresponded to different clear-phase extracts, ranging from 1 mL to 100 mL, in different reverse-phase 10-g C18 cartridges and eluted with the new optimized MeOH elution volume, in this case 200 mL. Subsequently, the hemolytic activity was determined in the different eluates obtained, in the same way as explained in Section 4.2.4. The retention volume (V_R_) of the analyte is defined as the inflection point of the curve where the retention capacity is reached [19]. As shown in Figure 2A, for the reverse-phase 1-g C18 cartridges, this point occurred at a 3.6 × 10^−4^ crude extract-to-adsorbent ratio, reaching a level of 20% ESP_effluent_ over ESP_crude_. This compares with the 1 × 10^−3^ clear-phase extract-to-adsorbent ratio that occurred in the new isolation strategy using the reverse-phase 10-g C18 cartridges (Figure 2A). This means that the sorbent retention capacity has improved by 1.77 times. The separation of the main target, the APDs, from the remaining compounds, such as carotenoids and fatty acids, was mainly due to the LLE that was applied as a first step. In this respect, LLE has been used for sample pre-concentration to detain marine lipophilic toxins [9]. Based on the data given in Figure 2A, no metabolites were detected in the effluent when a 2 × 10^−4^ clear-phase extract-to-adsorbent ratio was used. Therefore, the retention of APDs was favored because of the LLE used as a first step and the optimization of the clear-phase volume load to the 10-g C18 cartridge.

**Figure 7:**
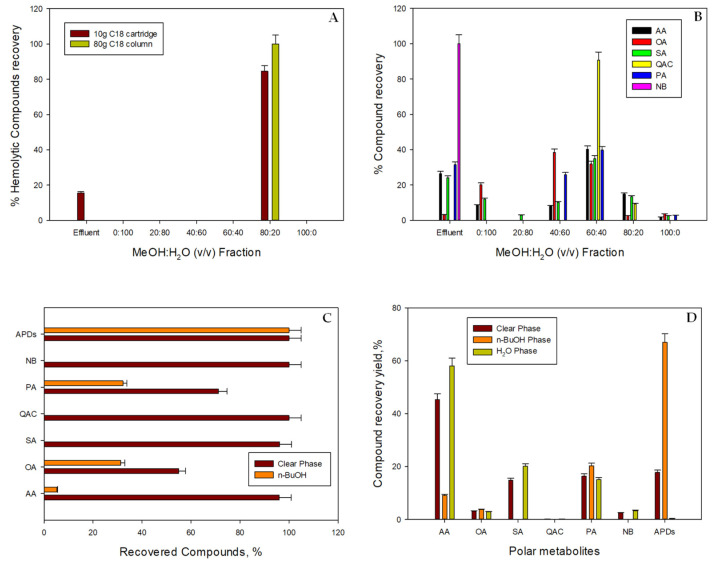
(**A**) Distribution of hemolytic compounds recovered by the fractionation process using the reverse-phase 10-g C18 cartridge (red bars) and the 80-g C18 column (green bars) and (**B**) the main polar metabolite groups recovered by the fractionation process using the 10-g reverse-phase C18 cartridges via the process outlined in Figure 4, Step 6, Option A. Percentages are relative to the content in the initial clear phase. (**C**) Distribution of the main metabolite groups over the two phases obtained from the two purification options outlined in Figure 4 (red bars, clear phase fractionation step procedure; orange bars, solvent-partitioning with n-BuOH). (**D**) Percentage of each compound with respect to the total of compounds present in the clear phase, n-BuOH phase, and H_2_O phase. AA: amino acids; OA: organic acids; SA: sugars; QAC: quaternary ammonium compounds; PA: polyhydric alcohols; NB: nitrogenous bases; APDs: amphidinols. Data bars are averages and vertical bars are standard deviations for duplicates samples.

To quantify (by NMR) the APDs contained in both the clear phase and in the different elution gradient fractions explained above, the clear-phase extract volume (0.1 mg·mL^−1^) that was loaded in the 10-g C18 cartridge was optimized assuming a 20% loss of APDs in the effluent. For this, 100 mL of clear phase was used. This clear-phase volume was first suspended in 570 mL of H_2_O to reach a 10% MeOH concentration. After following the fractionation and elution protocol, a sub-fraction of 6.5 (Figure 4, Option A) was found to be the most active with 84.48 ± 4.22% of the total APDs (Figure 7A). The remaining APDs (15.52 ± 0.77%) were detected in the effluent (not adsorbed), and a percentage lower than the 20% of APD losses was assumed for the clear-phase volume that was optimized for loading into the column.

Once the MeOH:H_2_O 80:20 (*v*/*v*) fraction was determined by hemolysis as the most active fraction, the process was scaled up to an 80-g C18 column. In this case, the optimization was performed to avoid the loss of APDs to the effluent. Then, 160 mL of clear phase was loaded into the column. This volume was suspended in 888.5 mL of H_2_O to again reach a 10% MeOH concentration. After carrying out the fractionation and elution protocol, a bio-guided search for hemolytic activity revealed that only the MeOH:H_2_O 80:20 (*v*/*v*) fraction was active, having an increased performance compared to the direct fractionation process and the clear-phase fractionation process using the 10-g C18 cartridge. APDs were recovered in Stream 6.5 with a recovery percentage of 100% ± 5%, and no APDs were found in the effluent (not adsorbed) (Figure 7A). This distribution pattern is an improvement on the patterns obtained in Section 2.5.1, as the APDs were concentrated in a clear-cut way in just one fraction.

#### 2.5.3. NMR-Based Metabolomics Approach to Assess the APD Purification Strategies

A deeper NMR-based metabolomics analysis approach was used to assess the processes described in Figure 4 (Option A of Step 6 and Option B), the aim being to fractionate the clear phase. The focus was on streams and fractions containing APDs. Figure 7B shows the compound recovery percentages from the Option A fractionation relative to the compound content in the crude MeOH extract (Figure 4, Step 2). The only fraction that recovered APDs (Stream 6.5; MeOH:H_2_O 80:20 (*v*/*v*)) carried moderately small amounts of the compounds, AA, OA, SA, and QAC, all of them below 16%. In contrast, Stream 6.4 (MeOH:H_2_O 60:40 (*v*/*v*)), which recovered no APDs, swept along significant amounts of all the compound groups, except NB; the recovery percentages ranged from the 32% minimum for OA to the maximum of around 90% for QAC (Figure 7B). NB was completely recovered in the effluent. This pattern differs from that observed in the direct fractionation process (Figure 5B). In those results, because the amounts of loaded compounds were higher (i.e., carotenoids and fatty acids), they could presumably force other polar metabolites to desorb in a more aqueous eluted fraction. Indeed, the resolution of the SPE device was lower than that of the new disposable SPE used here (an 80-g C18 column). Briefly, the SPE device resolution improves the higher the number of theoretical plates obtained by decreasing the particle diameter [6,19]—being 20–35 µm in the 80-g C18 column compared to 40–63 µm in the 10-g C18 cartridge.g C18 cartridge.

The above results from Option A of the fractionation step (Figure 4) contrast with those obtained in Option B using a simple solvent-partition with n-butanol (Figure 4). From the total of compounds contained in the clear phase, one can see that all the APDs were partitioned in the n-BuOH fraction and only tiny amounts of AA, OA, and PA were detected in this fraction (Figure 7C); the remaining compounds were swept along in the H_2_O fraction, especially the 100% of QAC and NB that were not detected in the n-BuOH fraction (Figure 7C). Even though both options achieved complete recovery of all the APDs, Option B could be a rapid and advantageous methodology for recovering all the APDs in just one fraction (n-BuOH), in which the majority of the other polar metabolites were not swept along. While Option A requires large amounts of solvents and is time-consuming, Option B uses fewer materials and solvents, all of which are readily available and relatively inexpensive. Therefore, Option B might be technically more feasible and less time-consuming.

Option B is based on a recently described solvent-partitioning process that has proven highly effective at defatting and desalting [4], providing APDs in a clear-cut way in the n-BuOH fraction. Nonetheless, tiny amounts of fatty acids and carotenoids were found to be present in that fraction, along with significant amounts of AA, OA, PA, SA, QAC, and NB [4]. In the study presented here, the LLE process provided a suitable tool to effectively remove fatty acids and carotenoids from the streams feeding Options A and B before being treated. Secondly, even though other polar metabolite interferences were inevitable, their proportion (expressed as the relative percentage of each metabolite with respect to the total of compounds contained in each phase) in Option B was lower than in Option A (33% versus 81%, respectively) (Figure 7D). In particular, <22% of AA, OA, and PA were swept along in the n-BuOH. Briefly, the n-BuOH fraction carried 70% of the APDs that were purified three times more than those in the clear phase (Figure 7D).

#### 2.5.4. Evaluation of the Free Carotenoid Isolation and the Fatty Acid Purification

To separate the carotenoids and fatty acids contained in the dark phase obtained in Section 2.5.2, a second solvent-partitioning was carried out, coupled with saponification extraction (Figure 4, Step 3). Alkaline treatment is commonly used to release carotenoids from their naturally-occurring ester form [24]. In some cases, peridinin undergoes hydrolysis, losing acetic acid to convert into peridinol, and then, in the presence of fatty acids, converts it into its ester form [16]. Table 1 shows the results when saponification extraction was carried out at 5% KOH, resulting in 98% of the carotenoids being recovered. A closer inspection of Table 1 shows the different effects of saponification on carotenoid recoveries. The maximum carotenoid value obtained was 129. 58 ± 6.47% in the dark phase using 20% KOH. This result was higher than for the compounds present in the initial dark phase (Figure 4, Step 2). In contrast, no carotenoids were detected in the MeOH:H_2_O 30:70 *v*/*v* phase. This effect is due to the saponification step, which is commonly used for hydrolyzing carotenoid esters [25,26]. These esters would otherwise remain in the initial dark extract together with many other lipids. For the same mg KOH/mg SLs ratio, the fatty acids recovered in the form of soaps in the hydroalcoholic phase (higher solubility) were 20.24 ± 3.98%, meaning that this KOH value was not high enough to efficiently solubilize the fatty acid salts. Accordingly, the mg KOH/mg SLs ratio was increased and the liquid-liquid extraction step was repeated to determine whether more fatty acids could be recovered from the hydroalcoholic phase.

Although improved recovery of fatty acid salts was observed when saponification was carried out at 40% KOH in the hydroalcoholic phase, carotenoid degradation increased, with their recovery percentage in the dichloromethane (CH_2_Cl_2_) phase being 43% lower. A higher mg KOH/mg SLs ratio might enhance the recovery of fatty acids in the hydroalcoholic phase but, at the same time, the risk of carotenoid degradation in the CH_2_Cl_2_ phase would greatly increase.

From Stream 3.2, the overall free carotenoid and fatty acid recoveries were 129.58 ± 6.47% and 79.75 ± 3.98%, respectively, using an optimal KOH-to-SLs ratio (*w*/*w*) of 0.67 mg KOH/mg SLs (20% KOH). Stream 3.1 allowed us to recover 20.24 ± 3.98% of the fatty acid salts, facilitating their purification by adjusting the pH using HCL, followed by extraction by adding hexane at a 1:1 (*v*/*v*) ratio.

## 3. Conclusions

It was possible to successfully optimize a direct fractionation process using SPE to isolate the amphidinols produced by *A. carterae*; almost 80% of these were recovered from the MeOH:H_2_O 80:20 (*v*/*v*) and MeOH:H_2_O 60:40 (*v*/*v*) fractions. For the direct fractionation of the crude extract, a 12-mL load is needed, which is equivalent to a 3.4 × 10^−4^ mg _extract_/mg_adsorbent_ ratio. The overlapping of fatty acids in the same fractions was inevitably observed, but this was not the case for carotenoids. As an alternative, an approach coupling liquid-liquid extraction with SPE has been demonstrated for separating the APDs, carotenoids, and fatty acids. The clear-phase extract volume needed to ensure APD losses were below 5% was optimized and the adsorbent was scaled up 80-fold using 80-g reverse-phase C18 columns—this improvement led to a 100% recovery of the APDs in the MeOH:H_2_O 80:20 (*v*/*v*) fraction. For the LLE coupled with SPE in an 80-g reverse-phase C18 column, a 160-mL load of clear phase is needed, which is equivalent to 2 × 10^−4^ mg_extract_/mg_sorbent_. Indeed, the NMR-based metabolomic approach proved the high level of purity of the APD-enriched fractions, obtaining close to 70% by solvent-partitioning using n-butanol—a level never attained before.

## 4. Materials and Methods

### 4.1. The Microalgae and the Production of Biomass

Strain Dn241EHAU of the marine dinoflagellate microalgae, *Amphidinium carterae,* was used [27]. It was obtained from the microalgae culture collection of the Plant Biology and Ecology Department of the University of the Basque Country. The biomass used in this study comes from a long-term (>270 days) culture grown in a pilot-scale, LED-illuminated raceway photobioreactor [28]. Details regarding the operation mode and experimental approach have recently been published [29]. Harvesting was carried out once the cultures entered the stationary phase. The cell suspension samples were harvested on day 260 and were centrifuged at 1000× *g* (RINA model 100 U, 200 SM centrifuge). The cell suspension contained 5 × 10^6^ cell·mL^−1^ with a 0.6 g·L^−1^ biomass concentration. The obtained pellets were gently washed with distilled water. Lastly, the cells were re-pelleted, lyophilized, and stored at −22 °C so that they would be ready for use in the different analytical procedures and extraction methods.

### 4.2. Optimization of the SPE-Based Fractionation

#### 4.2.1. Hemolytic Activity

*A. carterae* Dn241EHU contains amphidinols (APDs) that exhibit hemolytic activity [13]. Their content in extracts can be expressed in terms of their hemolytic activity on erythrocytes from defibrinated sheep blood, as described elsewhere [29]. Briefly, EC50 values for *A. carterae* (i.e., the number of cells per well giving 50% hemolysis) and a saponin control were calculated from dose-response Hill curves. Saponin was supplied by Sigma Aldrich (47036, CAS n. 8047-15-2, Saint Louis, MO, USA) and the corresponding EC50 was 8.5 ± 0.6 × 10^6^ pg per well. An equivalent saponin potency (ESP) was expressed in terms of mg saponin per *A. carterae* biomass and was calculated by dividing the EC50 for saponin by the EC50 for *A. carterae*. Knowing the mg·cell^−1^ (1.2 × 10^−7^) and the crude methanolic extract mass (0.3 mg·mL^−1^) from the 0.5 mg·mL^−1^ biomass concentration in methanol, the ESP can be calculated, an is expressed as mg saponin per mg *A. carterae* crude methanol extract.

As described in Section 4.5 below, APDs can be detected and quantified by NMR analysis [13]. Briefly, the assignment of APD metabolites is possible with the help of 2D NMR experiments, HRMS, and tandem MS. The two-dimensional ^1^H–^13^C HMBC spectrum of the extract itself allows for the identification of key fragments that are common in most of the amphidinolides that are already known [13]. Since ESP is linearly correlated with the absolute integral of the peak at δ_H_ 5.07 ppm assigned to APDs, ESP can be used as a proxy of the levels of APDs in cells or extracts [13].

#### 4.2.2. Pre-Treatment of Biomass Using Cell Disruption Methods

Two cell disruption methods were tested for pre-treating the biomass: (i) ultrasound (UT) using a probe-type device (UP200S, Hielscher Ultrasonics™, Teltow, Germany) in cycles of 50% and with an amplitude of 80% (UT); and (ii) milling based on a mortar and pestle without alumina (MP). All the assays, pre-treated (UT and MP) and not pre-treated (CTRL), were performed with freeze-dried biomass (5-mg samples). The biomass was directly extracted in 10 mL of methanol and shaken at room temperature (22–25 °C) in a vortex mixer (for tubes) for 1 min and then centrifuged for 8 min at 2500× *g* (Heraeus Labofuge 2000, Osterode, Germany). The control (CTRL) consisted of biomass without pre-treatment. For the MP method, the biomass was first placed in a mortar, and then pestled. In the UT method, after being extracted with methanol, the samples were sonicated for different lengths of time ranging from 15–45 min. The hemolytic activity was determined in the resulting supernatants, as described above. The efficiency of each method in recovering APDs was assessed in terms of the hemolytic activity compared to the control.

#### 4.2.3. Optimization of the Biomass-to-Extractant Ratio

It has been recently demonstrated that only solvents with polarity indexes (PI) and Hildebrand solubility parameters (δ_T_) above ca. 6 and 20 MPa^1/2^, respectively, can extract APDs from *A. carterae* biomass [4,5,6,7,8,9,10,11,12,13,14,15,16]. Thus, based on those studies, methanol was chosen as the extractant for the biomass used. To optimize the biomass-to-MeOH ratio (*w*/*v*), different amounts of lyophilized biomass (ranging from 2 to 300 mg d.w.) were suspended in 20 mL of HPLC-quality MeOH to obtain ratios ranging from 0.1 to 15 mg·mL^−1^. The biomass–MeOH mixtures were sonicated for the time and selected as the optimum in Section 4.2.2. Each biomass-to-MeOH ratio was tested in triplicate. The hemolytic activity of each extract was analyzed in triplicate.

#### 4.2.4. Sorption Capacity of the Reverse-Phase C18 Cartridge: The Breakthrough Curve

The maximum amount of organic material that can be retained in the reverse-phase SPE device (i.e., the C18 cartridge) was determined. For this purpose, different volumes of the same crude MeOH extract obtained in Section 4.2.3, equivalent to extracted biomass amounts ranging from 0.02 to 45 mg, were prepared by diluting them with deionized HPLC-quality water to a final 10% methanol concentration [30]. To carry out the SPE step, 1-g reverse-phase C18 cartridges (Hypersep, 40–63 µm, 100 Å, Thermo Scientific, Rockwood, TN, USA) were previously conditioned and equilibrated with 10 mL of a 50% MeOH:H_2_O solution 1:1 *v*/*v* (Figure 3, Step 2). Each sample volume of the crude MeOH extract was then loaded into different 1-g reverse-phase C18 cartridges (Figure 3, Step 3). Sorption was performed with negative pressure using a vacuum collector (Supelco Visiprep^TM^ DL, 10–15 in-Hg, Saint. Louis, MO, USA). The vacuum intensity was adjusted to allow the desired flow (2.4 mL·min^−1^), which was below the maximum recommended by the manufacturer (3 mL·min^−1^). Once the sample volume was extracted, the cartridge was washed with 10 mL of HPLC-quality water to remove any salts retained in the sorbent. The elution step was carried out with 100% MeOH (Figure 3, Step 4). The hemolytic activity of the different methanolic eluates was analyzed to establish the breakthrough curve. Breakthrough curves show the loading behaviour of the target analyte to be removed from solution in a fixed bed and is usually expressed in terms of the ratio of effluent analyte concentration (*C*) to inlet analyte concentration (*C*_0_), then defined as the *C/C*_0_ ratio [6,8,10,21]. The concentration of APDs in the inlet (i.e., the crude methanolic extract loaded in the C18 cartridge) and the eluate were expressed in terms of ESP and named as ESP_crude_ and ESP_effluent_, respectively. Therefore, the ESP _effluent_/ESP_crude_ ratio which corresponds to the ratio of effluent analyte concentration (*C*) to inlet analyte concentration (*C*_0_) explained above and was used to measure the APDs concentration variation in the inlet to outlet solution. The breakthrough point was defined as the point when the ESP_effluent_-to-ESP_crude_ ratio dropped below 5%, the quantity of analyte not adsorbed being effluent. This point allowed us to determine the maximum extract amount that can be loaded in the C18 cartridge, resulting in APD losses below 5%. Next, the C18 cartridges that were operated up to the breakthrough point were eluted with four different volumes of MeOH 100% (4, 10, 15, and 20 mL) to determine the MeOH volume that causes complete desorption of the compounds adsorbed on the sorbent. Again, the hemolytic activity measurements were taken. All the adsorption assays were conducted in triplicate, and, in each, the hemolytic activity measurements were performed in triplicate.

#### 4.2.5. SPE-Based Fractionation

The reverse-phase C18 cartridges (1 g) were operated up to the breakthrough point, as described in Section 4.2.4, and sequentially eluted with different MeOH:H_2_O (*v*/*v*) mixtures at the following proportions: 100:0, 80:20, 60:0, 40:60, 20:80, and 0:100. The polarity indexes (PI) and Hildebrand solubility parameters (δ_T_) of the mixtures ranged from 10.2 to 6.6 and from 47.8 to 29.6 MPa^1/2^, respectively (Figure 3, Step 4). The elution volume (10 mL) of each mixture used was determined, as explained in Section 4.2.4. The different eluted fractions were collected, including the effluent (not adsorbed), and their hemolytic activity was determined. All the assays were conducted in triplicate, and, in each, the hemolytic activity measurements were performed in triplicate.

### 4.3. Scale-Up of the APD-Prioritized SPE-Based Fractionation

Two APD-prioritized fractionation approaches were explored for separating the three valuable families of compounds present in the crude extracts: APDs, carotenoids, and PUFAs. The scale of adsorbent was increased 10-fold and 80-fold using reverse-phase 10-g C18 cartridges (Hypersep, 40–63 µm, 100 Å, Thermo Scientific, Rockwood, TN, USA) and 80-g C18 packed columns (Spherical, 20–35 µm, 100 Å, Agela Technologies, Torrance, CA, USA), respectively. The first approach consisted of direct fractionation of the crude extract, as described in Section 4.2.5, increasing the crude methanolic extract volume needed 10-fold to ensure APD losses were below 5% (Figure 3). The second approach is based on a three-step sequential process: (i) liquid-liquid extraction (LLE) of the crude methanolic extracts, followed by SPE and fractionation, which was aimed at enhancing the purification of the APDs (Figure 4, Option A). To compare our results with other authors, the loaded volumes were optimized to assume close to 20% of APD losses in the 10-g C18 cartridge and 0% APD loss in the 80-g C18 column by detecting them in the effluent. For comparison purposes, a second APD purification strategy was employed based on solvent-partitioning with n-butanol; this was proven to be suitable for APD isolation (Figure 4, Option B) [4].

#### 4.3.1. The Direct Fractionation by SPE Approach

Direct fractionation using a 10-g reverse-phase C18 cartridge (Hypersep C18 10 g, Thermo Scientific) was assessed by loading an optimized volume of crude MeOH extract (0.5 mg·mL^−1^) (Figure 3, Step 3). The volume of crude MeOH extract was first diluted with distilled water until a 10% MeOH concentration was reached. Following this, column calibration (Figure 3, Step 2) was performed, and then the organic materials retained on the cartridge were eluted with different H_2_O:MeOH mixtures, as explained in Section 4.2.5 (Figure 3, streams from 4.1 to 4.6). In this case, solid-phase extraction was always conducted in darkness to avoid carotenoid degradation. All measurements were carried out in triplicates.

#### 4.3.2. The Liquid-Liquid Extraction Coupled with SPE Approach

In this approach, a liquid-liquid extraction (LLE) step was introduced just before the adsorption step (Figure 4). The novelty of this LLE is that it combines two procedures reported in the literature [4,31]. One of them was used to recover karlotoxins (polyketides similar to APDs) that extracted the crude methanolic extract with CH_2_Cl_2_ to remove most of the lipids (fatty acids and pigments) [31]. The other one used a solvent-partitioning method to isolate the amphidinols produced by *A. carterae* in a clear-cut way in the n-BuOH fraction [4]. The LLE was carried out as follows:

Briefly, 500 mL of CH_2_Cl_2_ and 250 mL of distilled water were added to 500 mL of crude MeOH extract (0.3 mg·mL^−1^) (Figure 4, Step 2). The mixture was vigorously stirred at 250 rpm for 30 min and left to decant overnight in a refrigerated chamber at 5 °C. Two immiscible phases formed, namely the dark phase (487 mL) containing CH_2_Cl_2_ (PI 3.1; 20 MPa^1/2^) and the clear phase (763 mL) containing MeOH:H_2_O 70:30 *v*/*v* (PI 7.7; 34.82 MPa^1/2^). Both were separated by decantation in a 2-L glass separating funnel. The dark phase carried the carotenoids and fatty acids, whereas the clear phase carried the APDs and the other polar metabolites (i.e., amino acids, AA; organic acids, OA; sugars, SA; quaternary ammonium compounds, QAC; polyhydric alcohols, PA; nitrogenous bases, NB). It is believed that the virtual absence of lipids and pigments in the clear phase improves the APD sorption capacity of the C18 sorbent.

The dark phase was expected to contain carotenoids and fatty acids; therefore, this phase was subjected to a saponification reaction with simultaneous liquid-liquid extraction using a MeOH:H_2_O (30:70 *v*/*v*) mixture (Figure 4, Step 3). Briefly, KOH, 50 mL of the MeOH:H_2_O solvent, and 50 mL of the dark phase were mixed and stirred magnetically for 30 min at 250 rpm. The amount of added KOH was calculated based on the saponifiable lipid (SL) concentration in the dark extract. Thus, different assays were carried out by varying the KOH concentration from 5% to 80% *w*/*w* (mg KOH/mg SLs). Saponification was performed at 25 °C. Two immiscible new phases formed, namely the CH_2_Cl_2_ phase and the MeOH:H_2_O phase. These were separated by decantation in a 2-L separating funnel. While the CH_2_Cl_2_ phase (Figure 4, Stream 3.2) sweeps along the carotenoids, the saponifiable lipids are swept along by the MeOH:H_2_O phase (Figure 4, Stream 3.1). In a final step (Figure 4, Stream 3.1.1), the fatty acid salts contained in the hydroalcoholic phase were purified and recovered using 37% HCL to adjust the pH to 2. The fatty acids were then extracted by adding hexane at a 1:1 (*v*/*v*) ratio. The hexane phase was separated by decantation and evaluated for fatty acids, as explained elsewhere [4]. The recovery yields were calculated as the percentage of carotenoid and fatty acid contents with respect to the compounds present in the initial dark phase, which was obtained from Figure 4, Step 2.

After the LLE step in Figure 5, different volumes of clear phase were loaded into 10-g C18 cartridges (Figure 4, Step 5, Option A) to determine the breakthrough point volume and therefore establish the maximum extract amount that can be loaded in the 10-g C18 cartridge, leading to APD losses below 5%. Elution was carried out with the optimized volume of MeOH (200 mL).

The optimized clear phase volume was diluted with distilled water until a 10% (*v*/*v*) MeOH proportion was reached. Once again, column equilibration (Figure 4, Step 4, Option A) was performed before loading the methanolic extract. The sorption and fractionation steps were carried out as explained in Section 4.2.4 and Section 4.2.5 (Figure 4, Step 5 and Streams 6.1 to 6.6, Option A). Fractionation was performed using different C18 sorbent masses (i.e., 10 g and 80 g). The hemolytic measurements of the resulting water–methanol fractions were carried out in triplicate.

As an alternative, a purification strategy was applied on the clear phase using a simple solvent-partition with n-butanol (Figure 4, Option B). In short, 100 mL of clear phase was transferred into a flat-bottomed balloon and evaporated on a rotary evaporator to remove the solvent. The dry residue was firstly re-suspended in 100 mL of H_2_O and 100 mL of n-butanol (n-BuOH); then, the mixture was stirred magnetically for 30 min at 250 rpm and left to decant, as explained above (at the beginning of this section). Two immiscible phases formed and the n-BuOH phase was analyzed to determine the APDs.

### 4.4. Analytical Procedures

The following techniques were used both in the lyophilized biomass and in the different resulting extracts. The carotenoid content and profile were determined using an HPLC photodiode array detector, as previously explained [32]. Direct transesterification was used to determine the fatty acid methyl esters content and profile using gas chromatography coupled to a flame ionization detector (Agilent Technologies 6890 N Series Gas Chromatograph, Santa Clara, CA, USA), as described earlier [33]. The measurement was carried out in duplicate. The localization of the bioactive fraction from the presence of APDs was conducted by assay testing the hemolytic activity in the different fractions, as described above.

### 4.5. NMR Analysis

The capability of the process described in Figure 4 for isolating and purifying the APDs, compared to that shown in Figure 3, was analyzed using a recent untargeted and rapid NMR-based metabolomics approach [13]. NMR metabolic profiles were recorded on a Bruker Avance III HD 600 spectrometer operating at a proton frequency of 600 MHz and using a 5-mm QCI quadruple resonance pulsed field gradient cryoprobe. Acquisition was carried out with rotation at 293 ± 0.1 K and using a NOESY pre-saturation pulse sequence (Bruker 1D noesygppr1d). Details regarding the analytical procedure and metabolite quantification have recently been explained [4]. The following metabolites were identified and quantified: (i) amino acids, AA (valine, isoleucine, leucine, threonine, alanine, proline, methionine, glutamate, glutamine, glycine, lysine, aspartate, tryptophan, tyrosine, phenylalanine, histidine); (ii) organic acids, OA (lactate, acetate, succinate, fumarate, formate); (iii) sugars, SA (β-galactose, β-glucose, α-glucose, α-galactose); (iv) Quaternary ammonium compounds, QAC (choline, betaine); (v) Polyhydric alcohols, PA (glycerol); (vi) Nitrogenous bases, NB (uracil, cytosine); and (vii) APDs.

Considering the aim of the APD isolation method, first it had to establish the amount of each fraction (crude MeOH extract, clear phase, dark phase, and n-BuOH phase) needed for screening the products by NMR. For this, different volumes of fractions were dried to obtain a final quantity in a range between 10–20 mg of extracts from the crude phase, or the other different phases, to ensure the presence of APDs and to validate the results. The measurements were carried out in triplicate. All the phases (i.e., crude MeOH extract, clear phase and dark phase, n-BuOH phase, and water phase) and fractions were analyzed after the elution step procedure. However, the focus was on those fractions whose δ_T_ met the APD extraction requirements as reported earlier [15] (i.e., the clear phase and MeOH:H_2_O fractions ranged from 60% to 80% MeOH, obtained after fractionation of this phase (Figure 4, Option A) and the n-BuOH phase (Figure 4, Option B); the crude MeOH extract and MeOH:H_2_O fractions ranged from 60% to 80% MeOH (Figure 3) given that these fractions could potentially carry the APDs.)

### 4.6. Statistical Analysis

Statgraphics Centurion XVII (version 17.2.04) statistical software (2014, Statpoint Technologies, Inc., Warrenton, VA, USA) was used for: (a) a significant difference analysis with a one-way analysis of variance (ANOVA) test and (b) a significant difference analysis with a multi-way ANOVA test.

## Figures and Tables

**Table 1:** Recovery percentages of the free carotenoids and fatty acids recovered by liquid-liquid extraction and the simultaneous saponification procedure outlined in Figure 4, Stream 3. Recovery yield (% d.w.): Percentage of carotenoids and fatty acids extracted with respect to the compounds present in the initial dark phase (Figure 4, Step 2).

	Fatty Acids (%, Recovery Yield)		Carotenoids (%, Recovery Yield)	
KOH (% *w*/*w*)	CH_2_Cl_2_ Phase	MeOH:H_2_O 30:70 Phase	CH_2_Cl_2_ Phase	MeOH:H_2_O 30:70 Phase
5% KOH	100 ± 5	-	98 ± 5	2 ± 0
10% KOH	91 ± 5	9 ± 0	127 ± 6	-
20% KOH	80 ± 4	20 ± 1	130 ± 6	-
40% KOH	60 ± 3	40 ± 2	86 ± 4	-
60% KOH	40 ± 2	60 ± 3	46 ± 2	-
80% KOH	19 ± 1	81 ± 4	6 ± 0	-

## Data Availability

Not applicable.

## Nomenclature

AA: amino acids; APDs: amphidinols; SPE: solid-phase extraction; V_B_: breakthrough volume; BuOH: n-butanol; C18: silica-based octadecyl bonded phase; CH_2_Cl_2_: Dichloromethane; CRs: Carotenoids; CTRL: control extraction in absence of a pre-treatment; EC50: the number of cells per well giving 50% hemolysis; ESP: hemolytic activity of a given extract or fraction measured as equivalent saponin potency; FAs: Fatty acids; H_2_O: water; LLE: liquid-liquid extraction; MeOH: methanol; MP: pre-treatment with mortar and pestle without alumina; NB: nitrogenous bases; NMR: nuclear magnetic resonance; OA: organic acids; PA: polyhydric alcohols; PI: polarity index; PUFAs: polyunsaturated fatty acids; QAC: quaternary ammonium compounds; V_R_: retention volume; SA: sugars; UT: pre-treatment with ultrasound; δ_T_: Hildebrand solubility parameter.
